# The archibiome: extending engineered living materials to multiscale built environments

**DOI:** 10.3389/fsysb.2026.1943876

**Published:** 2026-09-28

**Authors:** A. Sandak, W. Pajerski, A. Gubenšek, K. Butina Ogorelec, A. Černoša

**Affiliations:** 1 InnoRenew CoE, Andrej Marušič Institute, University of Primorska, Koper, Slovenia; 2 The Faculty of Mathematics, Natural Sciences and Information Technologies, University of Primorska, Koper, Slovenia

**Keywords:** archibiome, built environment, engineered living materials, microbial ecology, microbiome, probiotic design, regenerative architecture, sustainable design

## Abstract

The integration of living systems into architecture is advancing rapidly through developments in biotechnology, materials science, and design approaches grounded in regenerative principles. Engineered Living Materials (ELMs) and living artefacts demonstrate how microbial systems can be embedded within materials to enable responsiveness, metabolic activity, and adaptive functionality. However, current applications remain largely confined to discrete material components, overlooking the complex and dynamic microbial ecosystems already present within buildings. This opinion paper introduces the concept of the archibiome as a framework for extending ELMs from isolated systems to interconnected architectural environments. The archibiome conceptualizes buildings as dynamic microbial ecosystems in which engineered systems, material interfaces, and native microbial communities interact across spatial and temporal scales. While this shift offers opportunities for adaptive and regenerative design, it also introduces challenges related to system stability, environmental variability, biosafety, and regulatory constraints. Addressing these challenges requires interdisciplinary approaches that integrate microbial ecology, materials engineering, architectural design, and regulatory science. Ultimately, the archibiome positions microbial life as a design variable, enabling a transition toward predictive, systems-level strategies for living architecture.

## 1 Introduction

The integration of living systems into design and architecture is rapidly advancing, driven by developments in biotechnology, synthetic biology, and materials science. It is also motivated by a broader shift toward rethinking human–nature interactions in the built environment and principles of regenerative architecture (Myers, 2012). Recent work emphasised the need for more unified frameworks to understand how biological and engineered systems connect across various scales and environments (Soga and Gaston, 2022). In this context, Engineered Living Materials (ELMs) and living artefacts have demonstrated how microbial systems can be incorporated into materials and designed objects to enable responsiveness, metabolic activity, and new forms of interaction. ELMs integrate living cells within engineered scaffolds to create adaptive and responsive functions (Rodrigo-Navarro et al., 2021; Sandak, 2023). Living artefacts extend this approach by incorporating living organisms as active participants in designed objects, where biological activity is not only used to produce material properties but also contributes to ongoing responsiveness, interaction, and new forms of human–living system relationships (Karana et al., 2020). Both approaches have expanded the role of biology in design, positioning living systems as functional and tuneable components.

Current applications of microorganisms in design are largely limited to discrete objects or material components. As a result, microbial life in design is often spatially bounded and considered at short temporal scales (Kembel et al., 2012). However, buildings are not isolated systems: they already contain diverse and dynamic microbial communities that interact with materials, environmental conditions, and human occupants, influencing factors such as environmental quality, material performance, and health (Bruno et al., 2022). This opinion paper proposes the archibiome as a conceptual framework for understanding and designing microbial systems at the scale of the built environment. We hypothesize that extending the principles of ELMs from separated material systems toward interconnected architectural ecosystems can enable new forms of regenerative and adaptive design, where microbial functions are considered as integral components of building performance. Through this framework, we present how engineered living systems, material interfaces, and native microbial communities can be understood as interacting components of a broader ecological design strategy.

## 2 From engineered living materials to living artefacts

Living artefacts have shown how biological processes can be incorporated into object-scale applications, where microorganisms act as dynamic media for interaction, aesthetics, or localized performance (Karana et al., 2020). In parallel, engineered living materials provide the technological foundation for such developments by enabling the programming and stabilization of biological functions within material substrates (Sandak, 2023; Kostiainen et al., 2024). However, these implementations remain largely confined to discrete artefacts or components. This limits their ability to engage with microbial systems as distributed ecological networks across built environments. Buildings and infrastructure already host complex microbial ecosystems shaped by ventilation, materials, occupancy, and environmental conditions (Bruno et al., 2022). Indoor microbiomes therefore play a meaningful role in air chemistry, surface interactions, and occupant exposure, yet are rarely treated as designable systems within architectural practice (Adams et al., 2016). Resilient microorganisms can be harnessed as living tools by architects and designers to develop materials, structures, and environments that actively support human health. This approach, known as probiotic design, integrates beneficial microbial ecosystems into building and urban contexts (Beckett, 2021). This gap between microbial reality and design practice highlights the need for a framework that integrates microbial ecology into architectural and infrastructural thinking.

## 3 The archibiome concept

The literature employs a range of partially overlapping terms to describe microbial communities in the built environment, including “building microbiomes”, “indoor microbiomes”, “façade microbiomes”, “microbiota of the built environment”, and broader descriptors such as “surface-associated microbial communities” or “urban microbiome” (Chen et al., 2023; Young et al., 2023; Bosch et al., 2024; McAlister et al., 2025). However, these existing terms are primarily descriptive: they characterize microbial assemblages as emergent outcomes of environmental and design conditions, without distinguishing whether or how these communities are intentionally shaped. Similarly, concepts such as the “urban microbiome” or “probiotic cities” typically operate at broader spatial scales and are often framed in descriptive or policy-oriented terms, emphasizing emergent microbial patterns at the city level. They therefore frame microorganisms as passive consequences of design rather than as active components within it. In contracts (not in contracts), we define the archibiome as the subset of the built-environment microbiome that is intentionally shaped through architectural and material interventions, and as an operational framework that links microbial dynamics to controllable design parameters across buildings and infrastructure. It includes both engineered microorganisms introduced through ELMs and native microbial communities whose recruitment, selection, and interactions are modulated through design strategies (Figure 1).

**FIGURE 1:**
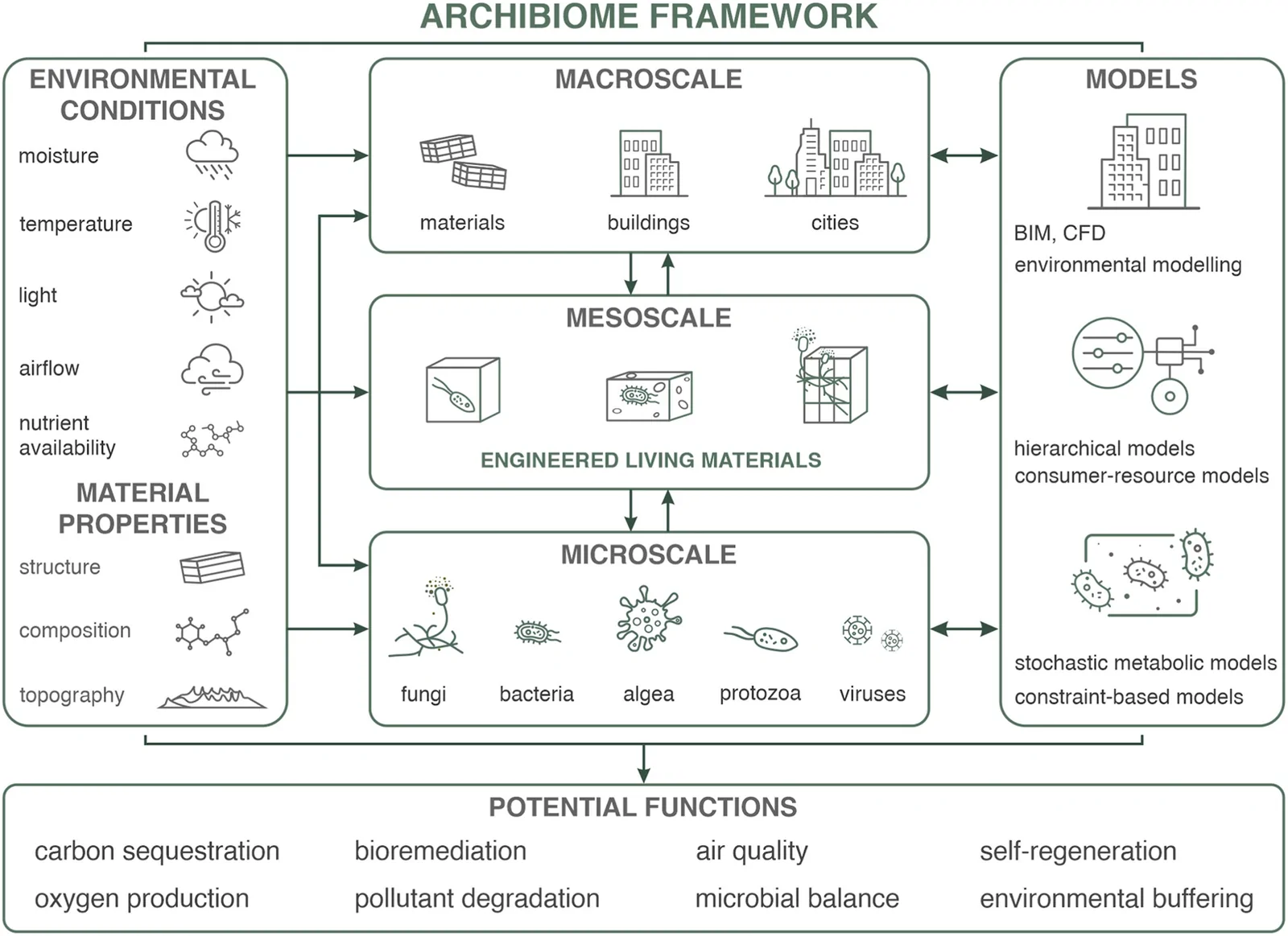
The archibiome framework for designing microbial systems at the scale of the built environment.

Conceptually, the archibiome treats the built environment as a multiscale, feedback-controlled system integrating microbial community dynamics, engineered materials, and environmental conditions. Microbial processes are coupled to architectural parameters (e.g., material composition, spatial configuration, moisture, light, and temperature) through feedback loops, in which system outputs influence future system states. This enables the prediction and regulation of system-level functions such as carbon sequestration, pollutant degradation, oxygen production, and self-regeneration.

Archibiome includes both engineered microorganisms introduced through ELMs and native microbial communities whose recruitment, selection, or ecological interactions are intentionally influenced by design strategies. It frames microbial communities as integral, designable ecosystems distributed within architectural and infrastructural systems (Robinson et al., 2024). Rather than focusing on isolated artefacts, the archibiome framework addresses how microbial processes are distributed and coordinated across spatial, temporal, and infrastructural scales, including their interactions with environmental conditions and human occupants. ELMs represent a key strategy for influencing the archibiome, offering tools to guide microbial ecosystem formation and to advance regenerative architectural approaches. ELMs act as enabling technologies through which microbial functions can be engineered, monitored, and controlled, while living artefacts represent localized implementations within a broader ecological system (Brown et al., 2016). The archibiome thus connects material-level innovation with building-scale performance, shifting the focus from isolated interventions to integrated ecological design. Importantly, the archibiome is not only defined by intentional design, but by the explicit integration of microbial dynamics with architectural control parameters. The archibiome framework focuses on how microbial communities are regulated through spatially distributed gradients and feedback mechanisms, including nutrient fluctuations, moisture regimes, material properties, and environmental exposure. ELMs provide the programmable physical substrate through which these gradients and feedbacks are implemented. Depending on their design, ELMs can perform biocatalytic (e.g., bioremediation, carbon sequestration), bioreceptive (e.g., protection, self-healing), and responsive (e.g., sensing, signalling) functions (Rodrigo-Navarro et al., 2021; Sandak, 2023). The intrinsic capacity of ELMs for self-regeneration and self-healing can substantially reduce maintenance requirements and material replacement over time, aligning with circular bioeconomy principles by lowering resource consumption and enhancing the environmental sustainability of building systems. This distinction positions the archibiome as an operational systems framework, in which microbial communities are not only selected but dynamically shaped through interactions between biological processes and architectural conditions. Microorganisms are treated as coupled components of a multiscale system, rather than as isolated or additive features within design. Table 1 provides a comparative summary of the conceptual and operational distinction between the existing concept of probiotic design and architecture and the proposed archibiome framework.

**TABLE 1:** Comparison between probiotic design and architecture and archibiome framework.

Probiotic design/architecture	Archibiome framework
Introduces beneficial microorganisms	Shapes microbial ecosystems through design parameters
Promotes a pro-microbial design	Treats microorganisms as designable and controllable components
Focuses on beneficial microorganisms	Includes both introduced and native microbial communities
Seeks to increase microbial diversity	Seeks to regulate, coordinate and stabilize microbial communities
Interventions at different scales: materials → buildings → urban planning	Integrates microbial processes across scales: materials → spaces → buildings → infrastructure
Emphasis on multispecies viability	Emphasis on system-level ecological function and feedback
Enabling technologies: Inoculation, probiotic materials, environmental monitoring	Enabling technologies: ELMs, real-time sensing, computational modeling, and feedback-driven control of microbial dynamics
Primarily empirical and descriptive	Integrates mechanistic and multiscale modeling
Limited emphasis on testable system-level hypotheses	Enables formulation of testable hypotheses linking design parameters to microbial dynamics and measurable functional outputs
Adopts known beneficial functions	Accounts for functional uncertainty using probabilistic modeling

## 4 Implications for sustainability, human health, and regulatory aspects

The importance of the archibiome is profound, particularly as modern humans spend up to 90% of their lives indoors (Dai et al., 2017). This prolonged exposure to the built environment means that indoor microbial communities, being an integral part of the exposome, play a critical role in shaping human health and wellbeing. Indoor air quality, exposed surfaces, and building materials shape the microbial landscape we interact with daily (Gilbert and Hartmann, 2024). While increasing evidence suggests associations between indoor microbial communities and human health, these relationships remain largely correlational, and further work is required to establish causal mechanisms and evidence-based design strategies. Designing indoor spaces with microbial ecosystems in mind therefore represents a promising but still emerging approach. This shift has direct implications for sustainability. Microbial communities contribute to processes such as carbon and nutrient cycling, pollutant degradation, and environmental buffering. Designing with these processes in mind could support new strategies for reducing resource consumption, improving indoor environmental quality, and enhancing resilience in the built environment (McAlister et al., 2025; Robinson et al., 2024). At the same time, the archibiome foregrounds human–microbial interactions, including exposure pathways and the broader concept of the human exposome, positioning microbial design within emerging health-oriented approaches (Dai et al., 2017).

Importantly, adopting an archibiome perspective also raises challenges that go beyond design and material integration. Engineering microbial ecosystems at the scale of buildings requires careful attention to long-term dynamics, system stability, and unintended ecological consequences. At the same time, it intersects with regulatory frameworks that are still evolving, particularly for products that intentionally introduce living microorganisms into built environments. The commercialization of products that create or modify archibiomes further complicates this landscape (Gilbert and Hartmann, 2024). Each ELM product is inherently unique, e.g., containing different organisms and having different functions and intended uses, meaning that one-size-fits-all regulatory pathways are insufficient. Regulatory uncertainty can slow the translation of research into practice, as extensive testing is required to demonstrate safety, efficacy, and ecological compatibility. In both the European Union and the United States, agencies such as the European Environment Agency (EEA) and the Environmental Protection Agency (EPA) enforce strict biosafety regulations that consider the potential spread of microorganisms beyond the targeted environment. If genetically modified microorganisms (GMMs) are involved, additional requirements address their origin, genetic modifications, and potential for horizontal gene transfer (Wesseler et al., 2023). While synthetic biology offers powerful tools for engineering ELM functionalities, its application in the building sector remains limited due to strict biosafety regulations restricting the use of genetically modified microorganisms outside controlled environments; therefore, current architectural implementations rely more feasibly on naturally occurring, extremotolerant microorganisms with broad environmental adaptability. Public perception is another critical factor. Concerns include uncontrolled microbial growth, allergenic responses, and survival of pathogenic organisms in sensitive environments such as hospitals or schools. These issues highlight the need for transparent communication and robust safety classification systems such as Qualified Presumption of Safety (QPS), Risk Group (RG) classifications, and FDA GRAS frameworks (Moreira et al., 2023). Ensuring long-term stability and safety of ELMs requires both physical and biological containment strategies. Hydrogels, thermogels, and synthetic or natural polymers can physically constrain microbial growth. The genetic and metabolic engineering of microorganisms limits their survival, proliferation, or gene exchange outside intended conditions (e.g., auxotrophy, kill-switches, or dependency on specific substrates). These approaches demonstrate the feasibility of creating living materials that are controllable, safe, and predictable (Sandak, 2023).

## 5 Discussion

Within the scope of ELM research, recent advances in synthetic biology, biointerfaces, and cell–material communication have enabled precise control of microbial and cellular functions across spatial and temporal scales. These developments provide the foundational capabilities required to translate ELMs from isolated functional materials into components of larger adaptive systems (Karana et al., 2020; Kostiainen et al., 2024). The archibiome builds directly on these capabilities by positioning such engineered functions within the broader context of built environment dynamics. While ELM research has primarily focused on optimizing cellular behaviour within defined material boundaries, the archibiome extends this challenge to include interactions between multiple engineered and native microbial populations distributed across architectural spaces (Soga and Gaston, 2022; McAlister et al., 2025). Importantly, the built environment is not a sterile context; introduced microorganisms will inevitably encounter resident microbial communities, leading to ecological interactions that may influence colonization, stability, function, and long-term performance of living systems. From a systems biology perspective, the archibiome can be formalized as a coupled multiscale dynamical system linking intracellular regulatory networks, interspecies interactions, and environmentally mediated boundary conditions. This provides a basis for developing predictive, testable models that connect biological function with architectural design decisions. At the microscale, stochastic or constraint-based metabolic models can describe microbial responses to environmental stimuli, including nutrient availability, stress, and signalling dynamics, and cooperation patterns (commensalism, mutualism) (Nadezhdin et al., 2020; Bosi et al., 2017). At the mesoscale, large hierarchical and consumer–resource models can capture competitive and cooperative dynamics governing community assembly and functional stability (Baldan et al., 2026). At the macroscale, Building Information Modelling (BIM) or computational fluid dynamics (CFD) can represent processes and environmental heterogeneity in the built environment (Joseph et al., 2025). These modelling layers can be integrated into a unified hybrid framework in which microbial dynamics are coupled to time-varying environmental fields defined by architectural parameters such as airflow, humidity gradients, surface chemistry, and material porosity. Such a framework enables the formulation of the built environment as a feedback-controlled ecological system, where environmental regulation and microbial activity co-determine system behaviour. Model parameterisation can be achieved through integration of multi-modal datasets, including metagenomics, metabolomics, and real-time environmental sensing, enabling data-assimilative calibration of mechanistic models of the environment (Munoz Briones and Brubaker, 2025). Iterative coupling between simulation and experimental validation might further allow hypothesis testing and model refinement. Within this framework, control-theoretic approaches may be employed to design architectural interventions that steer microbial community dynamics toward desired functional states, such as enhanced resilience, pollutant degradation, or stable beneficial colonisation. Extending such mechanistic models to built environments would enable predictive simulation of microbial ecosystem assembly, stability, and function across spatially distributed architectural systems, providing a quantitative foundation for archibiome-informed design and optimisation.

A central implication of this perspective is the need to move from single-material performance metrics toward system-level functional criteria. In ELM research, performance is often evaluated in terms of localized outputs such as self-healing, sensing, or catalysis (Rodrigo-Navarro et al., 2021; Karana et al., 2020; Liu and Xu, 2020). In contrast, the archibiome emphasizes emergent functions at the scale of buildings, including the shaping of microbial community dynamics, pollutant remediation, and the capacity of microbial ecosystems to influence local environmental conditions through chemical and ecological processes (Rodrigo-Navarro et al., 2021; Kostiainen et al., 2024; McAlister et al., 2025). Emerging research projects illustrate how the archibiome concept can be operationalized in practice. The ARCHI-SKIN project develops living coating systems based on the biofilm-forming fungus *Aureobasidium pullulans*, designed to protect building materials through self-regenerating and metabolically active layers. In this context, microbial performance is not only determined by species selection, but by the interaction between substrate properties, nutrient availability, and environmental conditions, highlighting the role of material–microbe–environment feedbacks. Complementary to this approach, the REMEDY project explores the integration of designed microbial consortia as “living inks” for architectural surfaces, combining functions such as carbon sequestration, pollutant remediation, and microbial balance with spatial patterning and digital fabrication strategies. The archibiome framework enables the formulation of explicitly testable design hypotheses by linking architectural parameters to microbial community dynamics and measurable functional outputs. For example, in the context of living active inks developed for architectural surfaces, which target carbon sequestration (up to 15 kg CO_2_·10 m^-2^ year^-1^), oxygen production via photosynthesis (up to 10 kg O_2_·10 m^-2^ year^-1^), self-regeneration (healing distance up to 2 mm), and bioremediation (up to 140 kg VOCs·air m^-3^·year^-1^), it can be hypothesized that spatial variation in environmental exposure, such as light intensity, moisture availability, temperature, and airflow regimes, will differentially modulate microbial metabolic activity and community composition across building surfaces. This hypothesis can be operationalized by predictive models of microbial growth and metabolite exchange with computational fluid dynamics simulations of airflow, light distribution, and humidity gradients, enabling prediction of spatially resolved performance metrics (Bosi et al., 2017). Empirical validation can then be achieved through meta-omics data, combined with *in situ* measurements of CO_2_ uptake, O_2_ production, and VOC degradation under both controlled and natural weathering conditions. Such an approach directly links design variables (e.g., façade orientation, material porosity, and environmental buffering capacity) with quantifiable ecological and functional outcomes, supporting iterative refinement of both models and archibiome-informed design strategies. Presented examples demonstrate how the archibiome extends beyond the introduction of beneficial microorganisms toward the system-level design and coordination of microbial ecosystems, supported by tools such as time-lapse imaging, computational modelling, and multimode microscopy and spectroscopy (Malat et al., 2026; Černoša et al., 2026). They illustrate how microbial communities can be distributed, monitored, and tuned across material and architectural scales, enabling the formulation of predictive and testable design strategies within the built environment. Operationalizing the archibiome concept further requires the definition of quantitative descriptors of system-level behaviour. Potential metrics include spatial patterns of microbial diversity and stability across building zones, rates of metabolite production or transformation (e.g., volatile compound degradation, carbon sequestration, and oxygen production), and surface colonization dynamics over time (self-healing and self-regeneration capacity). These indicators could be integrated into multi-scale performance frameworks, linking microscale biological activity with macroscale environmental outcomes. Such an approach would enable comparison, optimization, and validation of archibiome-based design strategies across different materials and environmental conditions (Song et al., 2014).

Translation of ELMs into built environment applications also raises significant engineering and lifecycle challenges. These include maintaining genetic and functional stability over time, ensuring reproducibility across production batches, and managing interactions between engineered systems and native microbial communities in open environments (Kostiainen et al., 2024; Liu and Xu, 2020). Such challenges are amplified when moving from contained material systems to architectural contexts, where microbial interactions, environmental variability, and feedback loops become more complex and less predictable (Bosi et al., 2017). Equally important are biosafety and regulatory considerations, which are explicitly highlighted as priorities within ELM research (Purnhagen and Wesseler, 2026). Deployment at the scale of buildings introduces additional uncertainty regarding containment, horizontal gene transfer, and long-term ecological effects. Engineered living systems require adaptive regulatory frameworks that account for their inherent variability and context-dependent behaviour (Wesseler et al., 2023). The archibiome framework reinforces this need by highlighting the expansion of biological interactions across spatial and temporal scales beyond individual material units. Public perception and societal acceptance also remain critical barriers to implementation. While ELMs are increasingly recognized as promising tools for sustainable design, their integration into everyday environments may raise concerns related to microbial exposure, health risks, and environmental control (Song et al., 2014; Wilssens et al., 2025). Addressing these concerns requires not only technical containment strategies such as hydrogels or polymer matrices (Rodrigo-Navarro et al., 2021), but also transparent communication and participatory approaches to design and deployment. Finally, the archibiome suggests new directions for ELM research itself. Rather than treating engineered living systems as isolated functional entities, future work may increasingly focus on their integration into distributed networks of interacting biological and material components. Realizing this vision requires close integration between materials engineering, microbial ecology, architectural design, and regulatory science. It includes the development of predictive models for multi-site microbial dynamics, scalable fabrication strategies for spatially heterogeneous ELM deployment, and design principles for maintaining functional stability across environmental gradients. Therefore, the archibiome does not diverge from the ELM field but extends its conceptual and operational boundaries. It highlights how advances in ELMs, synthetic biology, and systems biology can converge to support the development of adaptive, multifunctional built environments. Ultimately, the archibiome positions microbial ecology not as a byproduct of the built environment, but as a design variable that can be systematically modelled, tested, and controlled, marking a shift from descriptive to predictive and engineering-driven approaches to living systems in architecture.

## Data Availability

The original contributions presented in the study are included in the article/supplementary material, further inquiries can be directed to the corresponding author.
